# Glances at the Hospitals

**Published:** 1905-01-14

**Authors:** 


### GLANCES AT THE HOSPITALS.

MONTGOMERYSHIRE INFIRMARY.

The total income was £680, and the expenditure was £575. The cost of each in-patient was £6 6s., and the cost per head per week of patients and staff was 15s. 2¼d. The Annual Ball produced £66, and two concerts nearly £24. There were 10 patients in the wards at the beginning of the year, and 77 were admitted during it, making a total of 87. Of these, 33 were discharged recovered; 21 were discharged relieved; 13 for other reasons; 10 died, and 10 remained under treatment. A complete register of the in-patients is given in the report, and this register shows the age, sex, disease, number of days in hospital, and result of each patient's treatment. In a small hospital this is the best method of supplying the information. The average duration of each patient's stay in the wards was upwards of 36 days, which is a very high average.

